# The genus *Lochmaea* Weise, 1883 in Taiwan: results of taxonomic expeditions by citizen scientists (Coleoptera, Chrysomelidae, Galerucinae)

**DOI:** 10.3897/zookeys.856.30838

**Published:** 2019-06-17

**Authors:** Chi-Feng Lee

**Affiliations:** 1 Applied Zoology Division, Taiwan Agricultural Research Institute, 189 Chung-Cheng Road, Wufeng, Taichung 413, Taiwan Applied Zoology Division, Taiwan Agricultural Research Institute Taichung Taiwan

**Keywords:** Alpine, Ericaceae, host plants, leaf beetles, taxonomic revision

## Abstract

More than 520 specimens of the chrysomelid genus *Lochmaea* were available for study as the result of collecting efforts by citizen scientists. Taiwanese species of *Lochmaea* can be separated into two species groups based on presence or absence of hind wings. The *Lochmaealesagei* group (winged) contains *L.lesagei* Kimoto, 1996 and *L.tsoui***sp. n.** The *L.smetanai* group (wingless) contains *L.smetanai* Kimoto, 1996, *L.cheni***sp. n.**, and *L.jungchani***sp. n.** Members of the *L.smetanai* group inhabit alpine microhabitats and are the only wingless galerucines in Taiwan that occur in harsh environments, as is the case with most brachelytrous Chrysomelidae.

## Introduction

The genus *Lochmaea* Weise belongs to the Galerucini (Beenen 2010) based on antennal insertions that are close or equal to the anterior margins of the eyes. This genus is easily distinguished from similar genera such as *Galerucella* Crotch, 1873, *Mimastracella*Jacoby, 1903, and *Pyrrhalta* Joannis, 1865 by the absence of hairs on the dorsum with exception of *Lochmaealimbata* by having erect hairs on the dorsal surface and presence of a longitudinal convexity along the lateral margin of each elytron. Five species have been recorded from Europe and East Asia by Wilcox (1971), including *L.caprea* (Linnaeus, 1758), *L.crataegi* (Forster, 1771), *L.joliveti* Cobos, 1955, *L.limbata* Pic, 1898, and *L.suturalis* (Thomson, 1866). *Lochmaeasetulosa* (Sahlberg, 1913) was transferred from *Galerucella* by Silfverberg (1974). Kimoto (1979) described a new species, *L.maculata* Kimoto, 1979 from India. *Lochmaeasingalilaensis*Takizawa (1990) was also described from India. Two additional species, *L.lesagei* and *L.smetanai*, were described from Taiwan by Kimoto (1996). Beenen (1996) regarded *L.joliveti* Cobos, 1955 as a junior synonym of *L.scutellata* (Chevrolat, 1840). *Lochmaeahuanggangana* Yang and Wang was described from Fujian, China (Yang et al. 1998). Bezděk (2004) removed *L.machulkai* Roubal, 1926 from synonymy with *L.crataegi* (Forster, 1771). One more species, *L.nepalica*, was described from Nepal by Medvedev (2005). Gök et al. (2006) regarded *L.setulosa* (Sahlberg, 1913) as a junior synonym of *L.limbata* Pic, 1898. In total, twelve species are, at present, recognized as valid.

Members of *Lochmaea* utilize members of Betulaceae, Salicaceae, Rosaceae, Fagaceae, Ericaceae, and Cucurbitaceae as host plants (Jolivet and Hawkeswood 1995). *Lochmaeasuturalis* is well-known and referred to as “heather beetles” due to its monophagous feeding habits on heather foliage, *Callunavulgaris* (L.) Hull (Ericaceae) ((Stephens, 1831), Cameron et al. 1944). But Waloff (1987) noted that *Ericacinerea* L., *E.tetralix* L., and various cultivated species of *Erica* may also be suitable hosts. *Crataegusmonogyna* Jacq. (Rosaceae) was determined to be the host plant of *L.limbata* (Gök et al. 2006) in Turkey.

**Figures 1.**
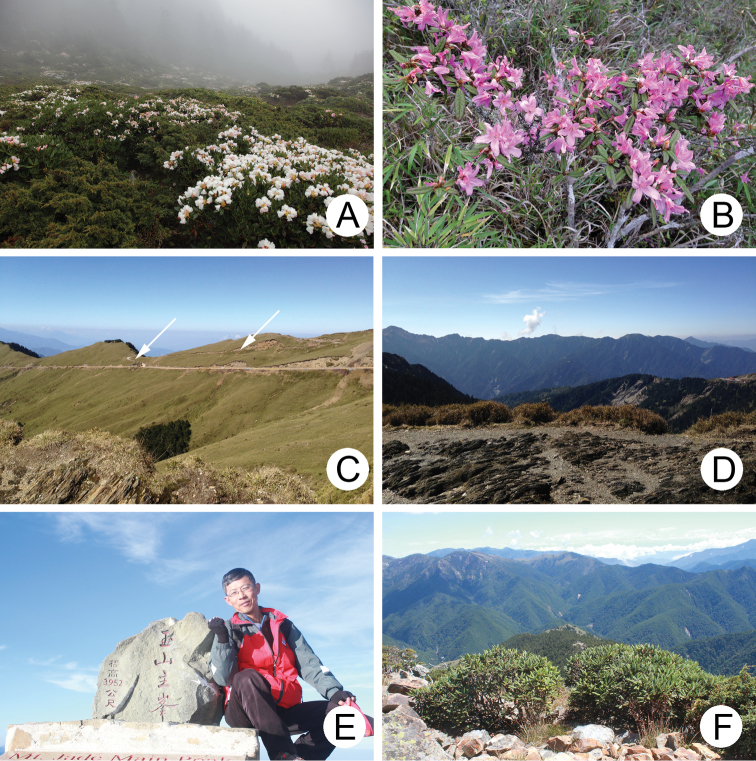
Field photographs. **A***Rhododendronpseudochrysanthum* blooming in June, Hsuehshan **B**R.rubropilosumHayatavar.taiwanalpinum blooming in June, Hehuanshan **C** Central Cross-Island Highway, arrows indicate road to top of Hehuanshan Main Peak **D** Wuling (武嶺), 3275 m, the highest spot at the Central Cross-Island Highway **E** Jung-Chan Chen, a member of the Taiwan Chrysomelid Research Team, at the top of Yushan Main Peak, 3952 m **F***R.pseudochrysanthum* , common at the top of Yushan East Peak.

No species of this genus was described from Taiwan until recently, when *L.lesagei* (winged) and *L.smetanai* (wingless) were described by Kimoto (1996) based on three specimens lacking biological information. The distribution and biology of Taiwanese species of Chrysomelidae have been investigated by members of the Taiwan Chrysomelid Research Team (TCRT) since 2005. As a result of their activities, larvae and adults of *Lochmaea* were found feeding on various species of *Rhododendron* (Ericaceae) at different localities. For example, populations have been discovered feeding on *R.formosanum* in Lupi (魯壁, 1450 m), *R.indicum* (Fig. 4E), and *R.hyperythrum* (Fig. 4C, D) in Lengshuikeng (冷水坑, 750 m), *R.pseudochrysanthum* in various localities above 2000 m (Fig. 4A, F). Moreover, wingless populations of *Lochmaea* were found only in alpine habitats above 3000 m. *Rhododendronpseudochrysanthum* are dominant plants in alpine regions and are the preferred hosts for wingless *Lochmaea* species. Members of this genus bloom and sprout during late spring (May and June) (Fig. 1A, B). In Taiwan, more than 250 mountains exceed 3000 m elevation, but only a few are easily accessable by hiking. Hehuanshan Moutain’s Main Peak (合歡山主峰, 3400 m) (Fig. 1C) and surrounding mountains (Eastern Peak, 3420 m; Western Peak 3145 m) can be accessed by walking only an hour since they are near the Central Cross-Island Highway (中橫公路) (Fig. 1D). Other mountains require days of climbing. Mr Jung-Chan Chen (陳榮章) (Fig. 1E), one member of TCRT, is capable of such hikes. For example, he took two days to reach the top of Yushan Main Peak (玉山主峰, 3952 m), but collecting was unproductive due to presence of only small host plants. He subsequently hiked for three days to reach the tops of Yushan East Peak (玉山東峰, 3869 m) (Fig. 1F), Yushan West Peak (玉山西峰, 3518 m), and Yushan North Peak (玉山北峰, 3833 m) and collected more than 30 specimens. These are in addition to material collected from various mountains by him during several years. As a result, species richness and distributions for each species of this genus can be accurately delimited based on robust sampling.

Beenen and Jolivet (2008) stated that most of brachelytrous chrysomelids (correlated with reduction of hind wings) occur in harsh environments including deserts, islands, and alpine regions. The proposed adaptive explanation for this condition is that in harsh environments energy has to be invested as efficiently as possible and investing in flight is maladaptive. *Lochmaea* is a unique genus in that it contains both winged species (*L.lesagei*) and wingless species (*L.smetanai*) in Taiwan. Thus, it is a good example to test whether the two species groups fit assumptions based on distributions in harsh habitats and correlated wing reduction.

## Materials and methods

Prior to the current study, a small number of specimens were collected using sweep nets and deposited at the Taiwan Agricultural Research Institute (**TARI**). Additional specimens collected using Malaise traps are deposited at the National Museum of Natural Science, Taichung (**NMNS**). Although adults are nocturnal, they stay on hosts during daytime where they can be collecting using sweep nets. They are active and walking during night time. Malaise traps can be effective, but beating host plants at night is the most effective way to collect adults, especially on plants with obvious feeding damage. In total, more than 520 specimens were available for this study using these collecting methods.

For rearing studies, larvae were placed in plastic containers (diameter 90 mm × height 57 mm) with cuttings from their host plants. When mature larvae began searching for pupation sites, they were transferred to other plastic containers of the same size but filled with moist soil (about 80% of container volume).

For taxonomic study, the abdomens of adults were separated from the forebody and boiled in 10% KOH solution, followed by washing in distilled water to prepare genitalia for illustrations. The genitalia were then dissected from the abdomen, mounted on slides in glycerin, and studied and drawn using a Leica M165 stereomicroscope. For detailed examinations a Nikon ECLIPSE 50i microscope was used.

At least two pairs from each species were examined to delimit variability of diagnostic characters. For species collected from more than one locality, at least one pair from each locality was examined. Length was measured from the anterior margin of the eye to the elytral apex, and width at the greatest width of the elytra.

Exact label data are cited for all type specimens of described species; a double slash (//) divides the data on different labels and a single slash (/) divides the data in different rows. Other comments and remarks are in square brackets: [p] – preceding data are printed, [h] – preceding data are handwritten, [w] – white label, [y] – yellow label, [g] – green label, [b] – blue label, and [r] – red label.

### Key to Taiwanese species of *Lochmaea*

**Table:** 

1	Elytral humerus and hind wing well developed (Fig. 2)	**2 (*L.lesagei* group)**
–	Elytral humerus and hind wing reduced (Figs 8, 11)	**3 (*L.smetanai* group)**
2	Median lobe symmetrical, with apex rounded (Fig. 3C); southern Taiwan	***L.lesagei* Kimoto**
–	Median lobe asymmetrical, with apex tapering (Fig. 6C); northern Taiwan	***L.tsoui* sp. n.**
3	Elytra green, with yellowish brown suture and lateral margins (Fig. 11A–C); median lobe parallel-sided (Fig. 12C); apical margin of abdominal ventrite V in females with median notch narrow and shallow (Fig. 12I)	***L.cheni* sp. n.**
–	Elytra entirely reddish brown or yellowish brown (Figs 8, 11D–F); median lobe apically tapering (Figs 9C, 13C); apical margin of ventrite V in female with median notch angular (Fig. 9I) or margined with longitudinal ridges (Fig. 13I)	**4**
4	Median lobe relatively broader, 5.7× longer than wide, elongate endophallic sclerite relatively longer, 0.7× as long as median lobe (Fig. 9C, D); apical margin of abdominal ventrite V in females with median notch angular (Fig. 9I)	***L.smetanai* Kimoto**
–	Median lobe relatively more narrow, 6.8× longer than wide, elongate endophallic sclerite relatively shorter, 0.5× as long as median lobe (Fig. 13C, D); apical margin of abdominal ventrite V in females with median notch narrow and margined with longitudinal ridges (Fig. 13I)	***L.jungchani* sp. n.**

## *Lochmaealesagei* species group

Members of this species group have well-developed elytral humeri and hind wings. Two species are recognized in Taiwan: *L.lesagei* Kimoto in South Taiwan and *L.tsoui* sp. n. in North Taiwan.

### Lochmaea lesagei

Taxon classificationAnimaliaColeopteraChrysomelidae

Kimoto, 1996

**Figures 2.**
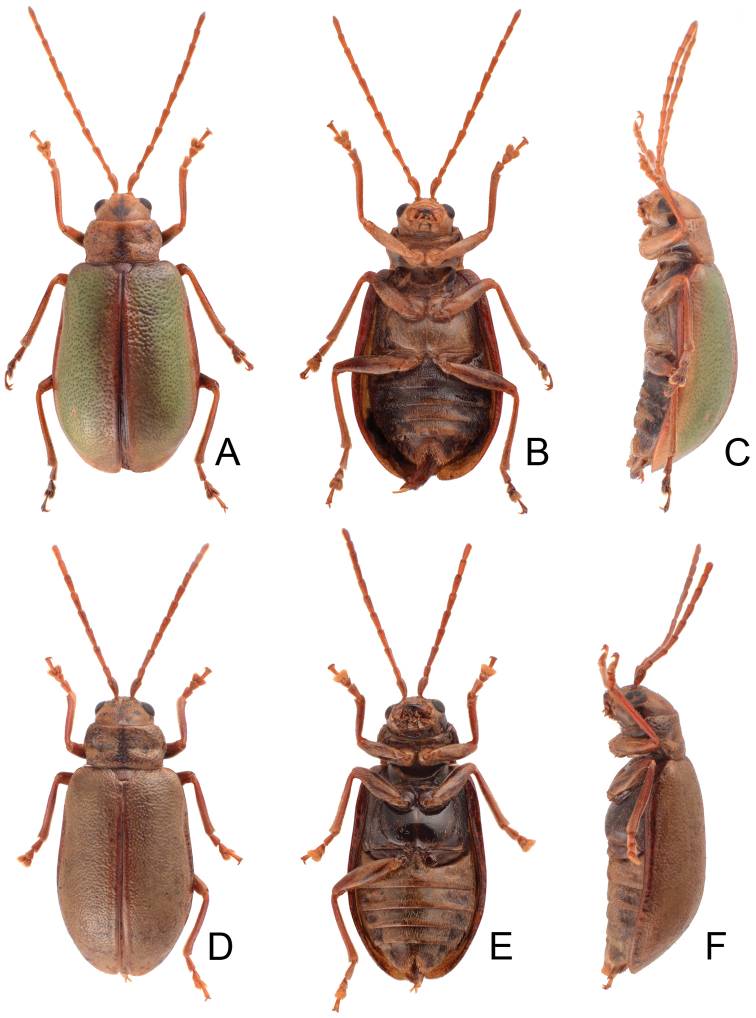
Habitus of *Lochmaea* species. **A***L.lesagei* Kimoto, female, dorsal view **B** Ditto, ventral view **C** Ditto, lateral view **D***L.tsoui* Lee, sp. n., female, dorsal view **E** Ditto, ventral view **F** Ditto, lateral view.

**Figures 3.**
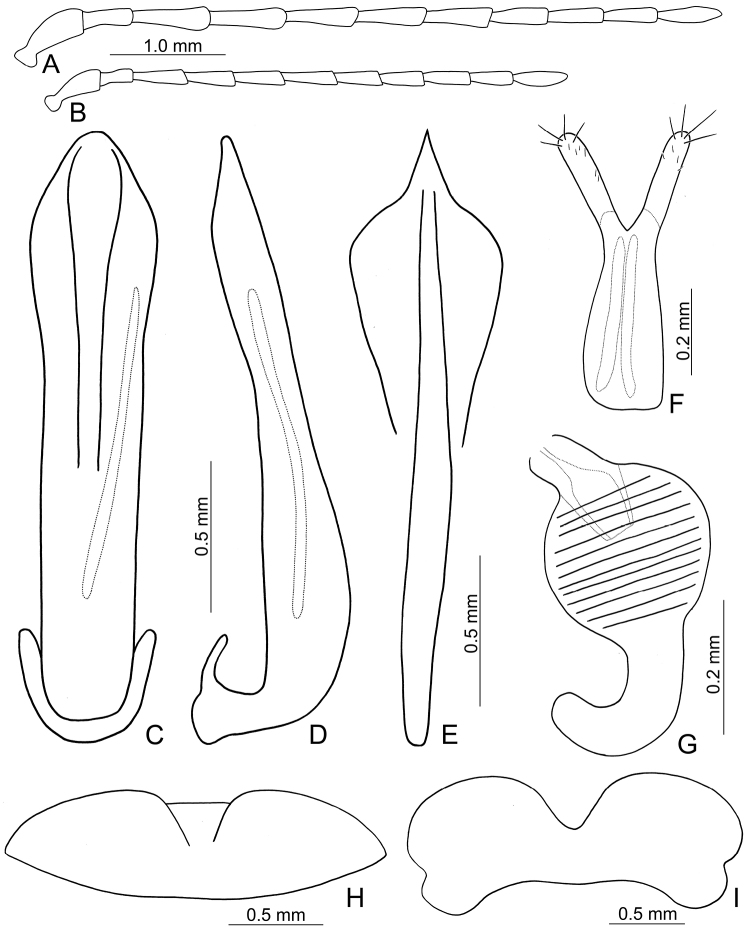
Diagnostic characters of *Lochmaealesagei* Kimoto. **A** Antenna, male **B** Antenna, female **C** Median lobe, dorsal view **D** Median lobe, lateral view **E** Abdominal ventrite VIII **F** Gonocoxae **G** Spermatheca **H** Abdominal ventrite V, male **I** Abdominal ventrite V, female.

**Figures 4.**
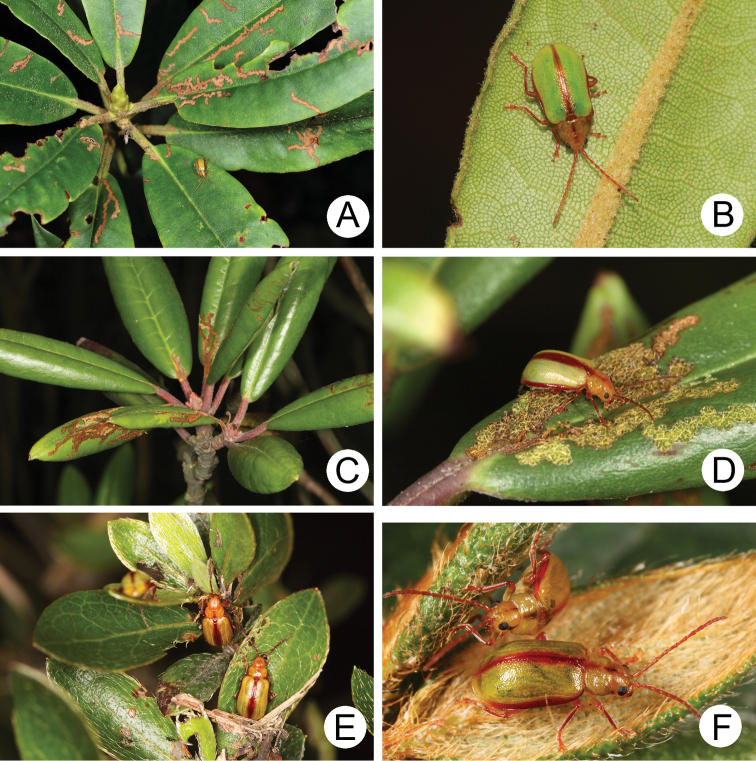
Field photographs. **A** Adult of *Lochmaealesagei* on the leaves of *Rhododendronpseudochrysanthum* at Tatachia **B** Same species also on *R.pseudochrysanthum* at Hsiangyangshan **C** Feeding marks on *R.hyperythrum* by *L.tsoui* sp. n. **D** Adult of *Lochmaeatsoui* sp. n. feeding leaves of *R.hyperythrum* at Lengshuikeng **E** Many adults of *L.tsoui* sp. n. were found on leaves of *R.indicum* at Lengshuikeng **F** Adults of *L.tsoui* sp. n. on the leaves of *R.pseudochrysanthum* at Hsuehshan.

Figs 2A–C , 3 , 4A, B

Lochmaea lesagei Kimoto, 1996: 32.

#### Type material.

**Holotype** ♂ (NMNS), labeled: “TAIWAN: Yushan / Nat. Park Mun-li / Cliff. 27.IV.90 / L. LeSage 2700 m [p, w] // Lochmaea / lesagei / Kimoto, n. sp. [h] / Det. S. Kimoto, 19 [p, w] // HOLOTYPE [p, r] // 2279-4 [p, w]”.

#### Other material examined (n = 109).

**Chiayi**: 2♂♂, 2♀♀ (TARI), Alishan (阿里山), 2400 m, 5–9.VIII.1981, leg. L.-Y. Chou & S.-C. Lin; **Hualien**: 1♂ (TARI), Tayuling (大禹嶺), 2550 m, 12–15.IX.1980, leg. K.-S. Lin & C.-H. Wang; 1♀ (TARI), same locality, 3.VIII.2015, leg. Uika; **Nantou**: 1♂ (TARI), Chilai South Peak (奇萊南峰), 3350 m, 23.VII.2017, leg. J.-C. Chen; 1♀ (TARI), Hehuanshan (合歡山), 3400 m, 6.IX.2017, leg. Y.-F. Hsu; 3♂♂ (TARI), Hsiaochilai (小奇萊), 3150 m, 23.IX.2016, leg. J.-C. Chen; 2♀♀ (TARI), Huakang (華崗), 2550 m, 12.IX.2010, leg. C.-F. Lee; 9♂♂, 9♀♀ (TARI), Kunyang (昆陽), 3050 m, reared from larvae, 23.VI.2009, leg. C.-F. Lee; 2♂♂, 2♀♀ (TARI), Tatachia (塔塔加), 2600 m, 20–21.VII.2009, leg. H. Lee; 2♂♂, 2♀♀ (TARI), same locality, 20.VII.2009, leg. C.-F. Lee; 1♂ (TARI), same locality, 21.IX.2009, leg. C.-F. Lee; 19♂♂, 19♀♀ (TARI), same locality, 9.IX.2015, leg. C.-F. Lee; 2♂♂, 1♀ (TARI), Tsuifeng (翠峰), 2300 m, 1–3.VIII.1981, leg. T. Lin & W.-S. Tang; 2♀♀ (TARI), same locality, 1–3.IX.1982, leg. L.-Y. Chou & K.-C. Chou; 1♂, 2♀♀ (TARI), same locality, 12–14.IX.1984, leg. K.-S. Lin & S.-C. Lin; 1♂, 1♀ (NMNS), Wushe (霧社), 1150 m, 15.X.2002, leg. C.-S. Lin; 1♀ (NMNS), Yuanfeng (鳶峰), 2750 m, 13.VI.-18.VII.2001, leg. C.-S. Lin & W. T. Yang; 1♂ (NMNS), same but with “7.VIII.-11.IX.2001”; 1♂ (NMNS), same but with “9.IV.-7.V.2002”; 1♂ (NMNS), same but with “9.VII.-13.VIII.2002”; 1♂, 1♀ (NMNS), same but with “13.VIII.-10.IX.2002”; 1♀ (NMNS), same but with “10.IX.-15.X.2002”; 1♀ (NMNS), same but with “15.X.-12.XI.2002”; 1♀ (NMNS), same but with “7.V.-11.VI.2003”; 1♂ (NMNS), same but with “8.VII.-5.VIII.2003”; 1♂ (NMNS), same but with “7.X.-4.XI.2003”; 1♂ (NMNS), same but with “5.X.-16.XI.2004”; 1♂ (NMNS), same but with “4.X.-8.XI.2005”; 1♀ (NMNS), same but with “21.IX.-17.X.2006”; **Pingtung**: 1♂ (TARI), Peitawushan (北大武山), 3050 m, 15.VIII.2016, leg. Y.-M. Weng; **Taichung**: 1♀ (TARI), Nanhutashan (南湖大山), 3700 m, 23.VII.2016, leg. J.-C. Chen; **Taitung**: 1♂, 5♀♀ (TARI), Hsiangyangshan (向陽山), 3600 m, 20.VI.2014, leg. J.-C. Chen; 1♂ (TARI), same but with “19.IX.2014”.

#### Diagnosis.

*Lochmaealesagei* Kimoto cannot be distinguished from *L.tsoui* sp. n. based on external morphology but it differs by the rounded apex of the symmetrical median lobe (Fig. 3C) (tapering apex of asymmetrical median lobe (Fig. 6C) in *L.tsoui* sp. n.), the acute apex of abdominal ventrite VIII in females (Fig. 3E) (rounded apex (Fig. 6E) in *L.tsoui* sp. n.), and northern Taiwan distribution (southern Taiwan in *L.tsoui* sp. n.).

#### Redescription.

Length 6.6–7.4 mm, width 3.3–2.9 mm. General color (Fig. 2A, C) yellowish brown to reddish brown; vertex and pronotum with median longitudinal dark band; each elytron green but with wide yellowish brown band along suture and lateral margin. Antennae filiform in males (Fig. 3A), length ratios of antennomeres I–XI 1.0 : 0.6 : 1.0 : 1.0 : 1.0 : 0.9 : 0.9 : 0.7 : 0.7 : 0.7 : 0.9, length to width ratios of antennomeres I–XI 2.7 : 3.0 : 3.3 : 3.4 : 3.6 : 3.6 : 3.6 : 3.5 : 3.7 : 3.6 : 4.0; much shorter in females (Fig. 3B), length ratios of antennomeres I–XI 1.0 : 0.5 : 0.9 : 0.8 : 0.9 : 0.9 : 0.7 : 0.7 : 0.7 : 0.7 : 0.9, length to width ratios of antennomeres I–XI 2.7 : 2.2 : 3.7 : 3.3 : 3.8 : 3.6 : 3.0 : 3.1 : 3.3 : 3.1 : 3.6. Pronotum transverse, 1.8× wider than long, disc with dense, extremely coarse punctures, and one pair of lateral depressions; lateral margins strongly narrowed basally; margins concave basally and apically. Elytra elongate and parallel-sided, 1.4× longer than wide; disc with random, dense, coarse punctures. Apical margin of abdominal ventrite V in males with median notch bearing short, longitudinal ridges along margin, concave between ridges (Fig. 3H). Ventrite V in females with deep, wide, median, rounded notch (Fig. 3I). Median lobe symmetrical, (Fig. 3C, D) slender, 6.4× longer than wide, parallel-sided from base to apical 1/3, broader towards 1/7, widest at apical 1/7, apex rounded; opening elongate, apically broader; in lateral view almost straight, strongly curved near base, apically narrowed from apical 1/7; internal sac with one elongate sclerite, 0.6× as long as median lobe. Gonocoxae (Fig. 3F) elongate, membranous except apical parts, with one pair of weakly sclerotized, elongate sclerites at base; apical parts elongate, bearing tiny, scattered setae and four long setae at apices. Ventrite VIII (Fig. 3E) longitudinal and well sclerotized; apex acute; abruptly broader at apical 1/5, spiculum long and wide. Receptacle of spermatheca (Fig. 3G) strongly swollen; pump slender and strongly curved; proximal spermathecal duct deeply inserted into receptacle, broad but short.

#### Host plants.

Ericaceae: *Rhododendronpseudochrysanthum* Hayata (Fig. 4A, B) and R.rubropilosumHayatavar.taiwanalpinum (Ohwi).

#### Biology.

Larvae appear when host plants begin sprouting. A number of young larvae (first-instar) were collected from *Rhododendronpseudochrysanthum* in Kunyang (昆陽) (3050 m), May 18, 2009 and transferred to the laboratory for rearing. Mature larvae burrowed into the soil and built underground chambers for pupation after seven days (May 25). Adults emerged from soil after 24 days (June 28). Twenty larvae emerged successfully as adults. From this sample, eighteen adults were identified as *L.lesagei* (winged) and the other two as *L.smetanai* (wingless). Adults appeared in the field from late June to October.

#### Distribution.

**Figure 5.**
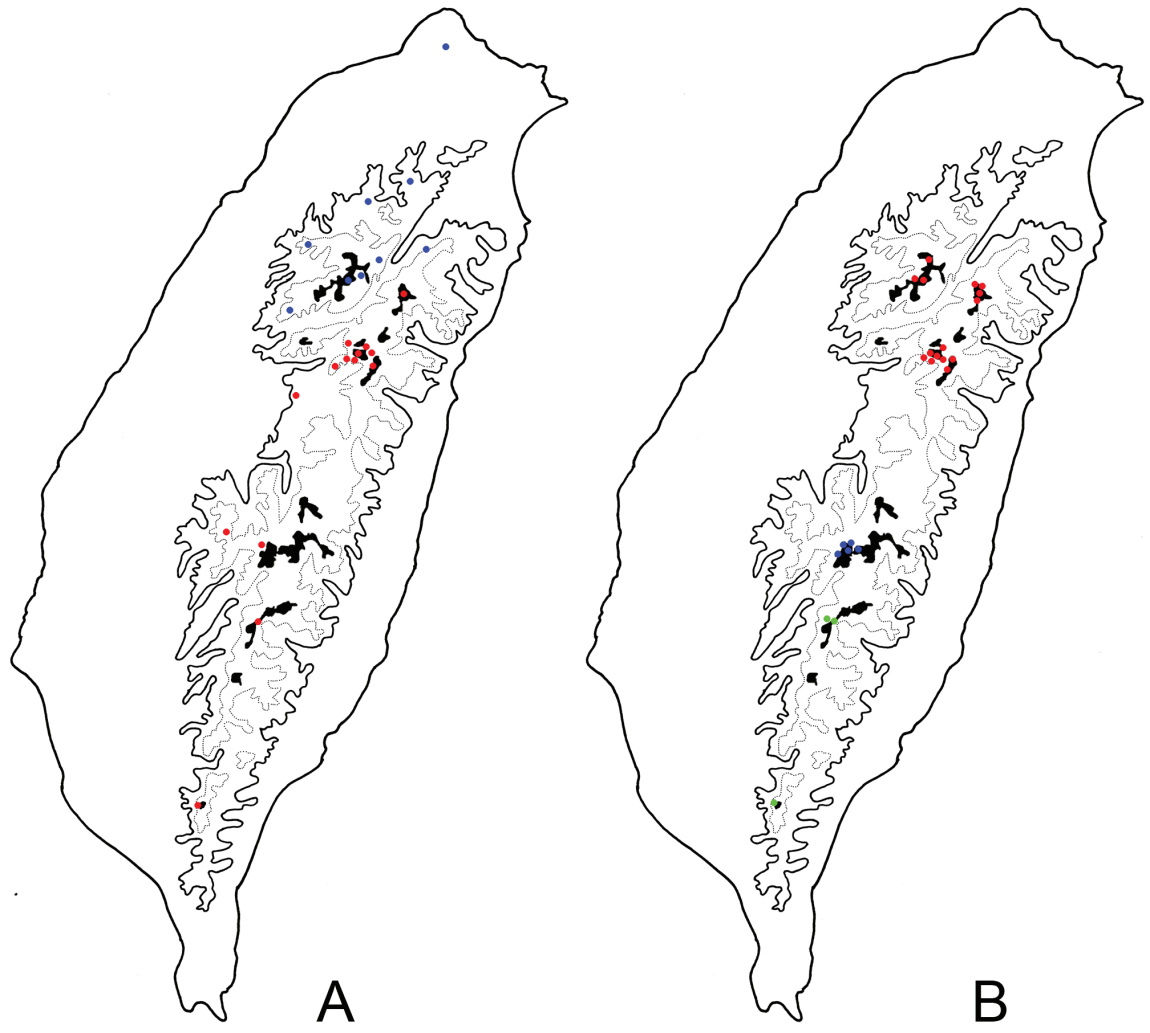
Distribution map of *Lochmaea* species, solid line: 1000 m, broken line: 2000 m, black areas: 3000 m. **A***L.lesagei* group. Key: Red Dots *L.lesagei* Kimoto Blue Dots *L.tsoui* sp. n. **B***L.smetanai* group. Red Dots *L.smetanai* Kimoto Blue Dots *L.jungchani* sp. n Green Dots *L.cheni* sp. n.

Southern Taiwan, including Nantou, Hualien, Chiayi, Pingtung, Taitung, and Taichung (only found in Nanhutashan (南湖大山)) Counties (Fig. 5A).

### Lochmaea tsoui sp. n.

Taxon classificationAnimaliaColeopteraChrysomelidae

http://zoobank.org/570DA489-1D13-4208-80D8-0D253EA53573

**Figures 6.**
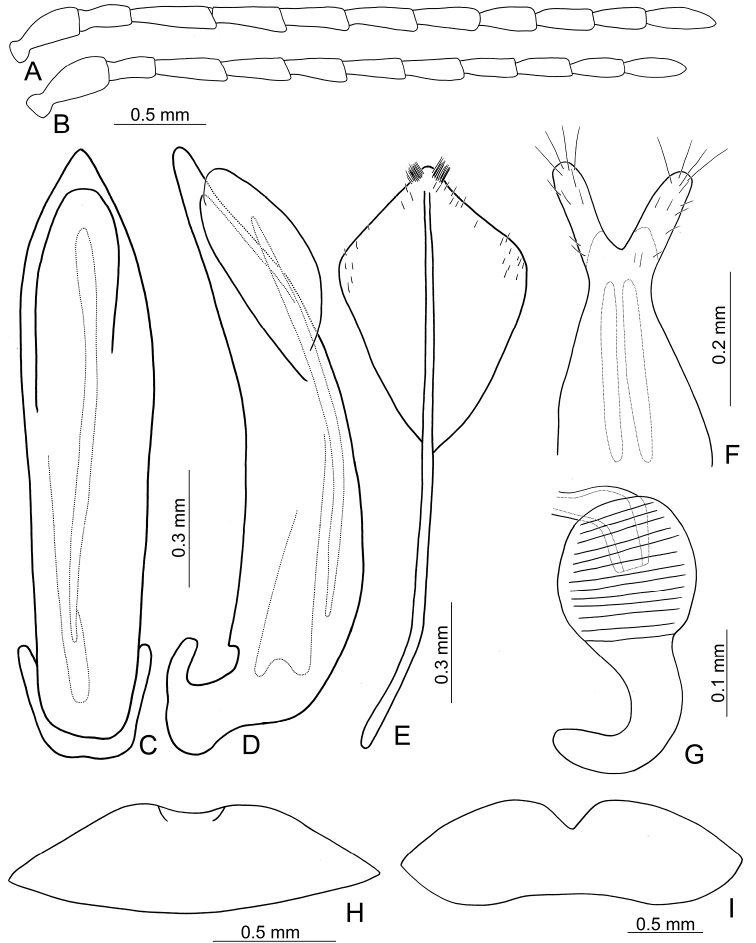
Diagnostic characters of *Lochmaeatsoui* Lee sp. n. **A** Antenna, male **B** Antenna, female **C** Penis, dorsal view **D** Penis, lateral view **E** Abdominal ventrite VIII **F** Gonocoxae **G** Spermatheca **H** Abdominal ventrite V, male **I** Abdominal ventrite V, female.

**Figures 7.**
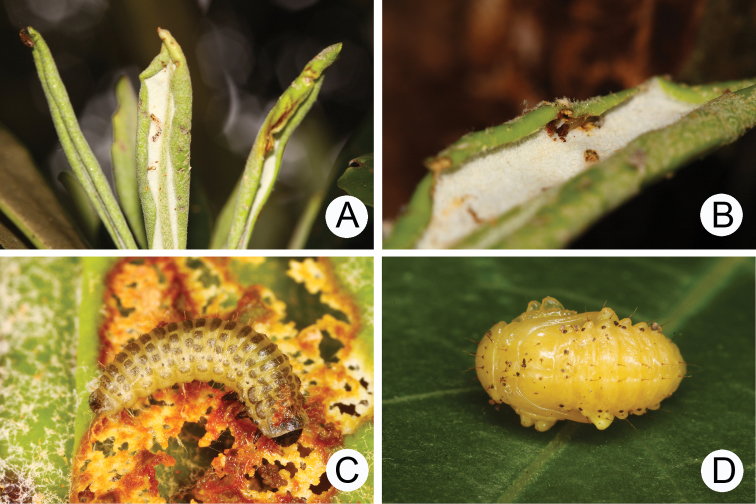
Ecological photography of *Lochmaeatsoui* Lee sp. n. **A** Feeding marks made by mining first-instar larvae **B** First-instar larva concealed inside coiled leaf **C** Third-instar larva **D** Pupa.

Figs 2A–2C , 4C–4F , 6 , 7

#### Type material

**(n = 84).** Holotype ♂ (TARI). **Hsinchu**: Lupi (魯壁), 1450 m, 26.VII.2008, leg. M.-H. Tsou. Paratypes. 5♂♂, 4♀♀ (TARI), same data as holotype; 4♂♂, 4♀♀ (TARI), same but with “20.VII.2008”; 1♀ (TARI), Kuanwu (觀霧), 2200 m, 6.XI.2009, leg. H. Lee; **Ilan**: 1♂, 1♀ (TARI), Tsuifenghu (翠峰湖), 1900 m, 3.VII.2010, leg. M.-H. Tsou; **Taichung**: 1♂ (TARI), Cika Lodge (七卡山莊), 2450 m, 30.IV.2012, leg. T.-H. Lee; 3♂♂, 1♀ (TARI), same locality, 3.IX.2014, leg. T.-H. Lee; 1♂ (TARI), Hsuehshan (雪山), 3850 m, 7.X.2011, leg. W.-B. Yeh; 1♂ (TARI), same but with “26.VI.2017”; 1♂ (TARI), same but with “15.VIII.2017”; 3♂♂, 3♀♀ (TARI), Kupo (哭坡), 2950 m, 2.IX.2014, leg. J.-C. Chen; 1♂ (TARI), Tahsuchshan (大雪山), 2550 m, 23.VII.2011, leg. J.-C. Chen; **Taipei**: 2♂♂, 7♀♀ (TARI), Lengshuikeng (冷水坑), 750 m, 26.V.2009, leg. J.-C. Chen; 9♂♂, 22♀♀ (TARI), same locality, 28.V.2009, leg. M.-H. Tsou; 3♂♂, 4♀♀ (TARI), same locality, 13.VI.2009, leg. H. Lee; **Taoyuan**: 1♂ (TARI), Lalashan (拉拉山), 1600 m, 30.X.2008, leg. S.-F. Yu.

#### Diagnosis.

*Lochmaeatsoui* sp. n. cannot be distinguished from *L.lesagei* Kimoto based on external morphology but differs by the tapering apex of the asymmetrical median lobe (Fig. 6C) (rounded apex of symmetrical median lobe (Fig. 3C) in *L.lesagei*), the rounded apex of abdominal ventrite VIII in females (Fig. 6E) (acute apex (Fig. 3E) in *L.lesagei*), and northern Taiwan distribution (southern Taiwan in *L.lesagei*)

#### Description.

Length 5.3–6.8 mm, width 2.7–3.3 mm. General color (Fig. 2D–F) yellowish brown to reddish brown; each elytron green but with wide yellowish brown band along suture and lateral margin. Antennae filiform in males (Fig. 6A), length ratios of antennomeres I–XI 1.0 : 0.7 : 1.1 : 1.0 : 0.9 : 0.9 : 0.9 : 0.8 : 0.8 : 0.7 : 0.9, length to width ratios of antennomeres I–XI 2.6 : 2.4 : 3.7 : 3.1 : 2.9 : 2.9 : 2.9 : 2.5 : 2.6 : 2.4 : 3.2; similar in females (Fig. 6B), length ratios of antennomeres I–XI 1.0 : 0.5 : 0.8 : 0.8 : 0.7 : 0.7 : 0.7 : 0.6 : 0.6 : 0.6 : 0.7, length to width ratios of antennomeres I–XI 2.4 : 2.1 : 3.2 : 3.0 : 2.6 : 2.7 : 2.7 : 2.8 : 2.9 : 2.9 : 3.2. Pronotum transverse, 1.8× wider than long, disc with dense, extremely coarse punctures, and one pair of lateral depressions; lateral margins strongly narrowed basally; margins concave basally and apically. Elytra elongate and parallel-sided, 1.4× longer than wide; disc with random, dense, coarse punctures. Apical margin of abdominal ventrite V in males with median notch, bearing short, longitudinal ridges along margin, shallow concave between ridges (Fig. 6H). Ventrite V in females with shallow, wide, median, angular notch (Fig. 6I). Median lobe (Fig. 6C, D) broad, 4.8× longer than wide, asymmetrical, left lateral margin straight, right lateral margin widest at apical 1/5, apically tapering; opening broad, located on right, starting from apical 1/12; in lateral view strongly curved, distinctly oblique; internal sac with one elongate sclerite, 0.8× as long as median lobe, one additional sclerite located near base of elongate sclerites, base wide and bifurcate, apically membranous. Gonocoxae (Fig. 6F) elongate, membranous except apical parts, with one pair of weakly sclerotized, elongate sclerites at base; apical parts elongate, bearing tiny, scattered setae and four long setae at apices. Ventrite VIII (Fig. 6E) longitudinal and well sclerotized; apex rounded; abruptly broader at apical 1/5, with paired cluster of long setae near middle, disc bearing tiny, scattered setae along apical margin; spiculum long and narrow. Receptacle of spermatheca (Fig. 6G) strongly swollen; pump slender and strongly curved; proximal spermathecal duct deeply inserted into receptacle, broad but short.

#### Host plants.

Ericaceae: *Rhododendronformosanum* Hemsl., *R.indicum* (L.) Sweet (introduced species) (Fig. 4E), and *R.hyperythrum* Hayata (Fig. 4C, D), and *R.pseudochrysanthum* Hayata (Fig. 4F).

#### Biology.

Different species of *Rhododendron* are available as food plants at different localities. A population feeds on *R.formosanum* in Lupi (魯壁, 1450 m), *R.indicum*, and *R.hyperythrum* in Lengshuikeng (冷水坑, 750 m), and *R.pseudochrysanthum* in various localities above 2000 m. First-instar larvae were collected in Lupi (魯壁, 1450 m) and transferred to the laboratory for rearing in April 4, 2009. They mined leaves (Fig. 7A), and some concealed themselves inside coiled leaves (Fig. 7B). Mature larvae (Fig. 7C) burrowed in soil and built underground chambers for pupation (Fig. 7D) after 15 days (April 19). Adults emerged from soil after 23 days. Adults appeared in the field from June to November.

#### Etymology.

This new species is named after Mr. Mei-Hua Tsou, a member of the TCRT and the first to collect this new species.

#### Distribution.

Northern Taiwan (Fig. 5A), including Taipei, Ilan, Taoyuan, Hsinchu, and Taichung Counties.

## *Lochmaeasmetanai* species group

Members of this species group have reduced elytral humeri and hind wings. Three species are recognized in Taiwan: *L.smetanai* Kimoto in northern Taiwan, *L.cheni* sp. n. in central Taiwan, and *L.jungchani* sp. n. in southern Taiwan.

### Lochmaea smetanai

Taxon classificationAnimaliaColeopteraChrysomelidae

Kimoto, 1996

**Figures 8.**
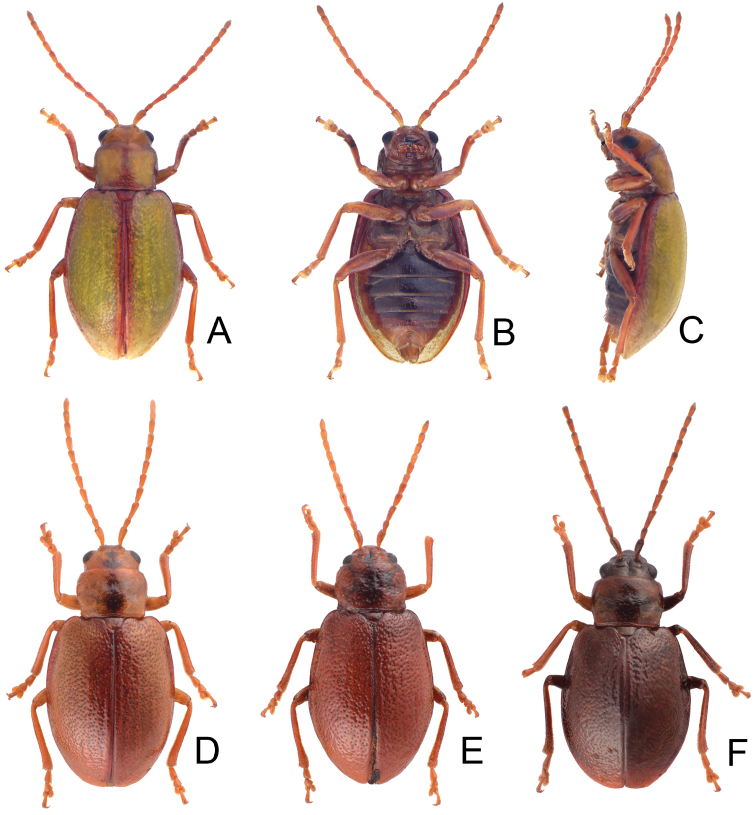
Habitus of *Lochmaeasmetanai* Kimoto. **A** Female, from Hehuan East Peak, dorsal view **B** Ditto, ventral view **C** Ditto, lateral view **D** Color variation, from Huhua Main Peak, dorsal view **E** Color variation, from Tsuichih, dorsal view **F** Color variation, from Hsiaochilai, lateral view.

**Figures 9.**
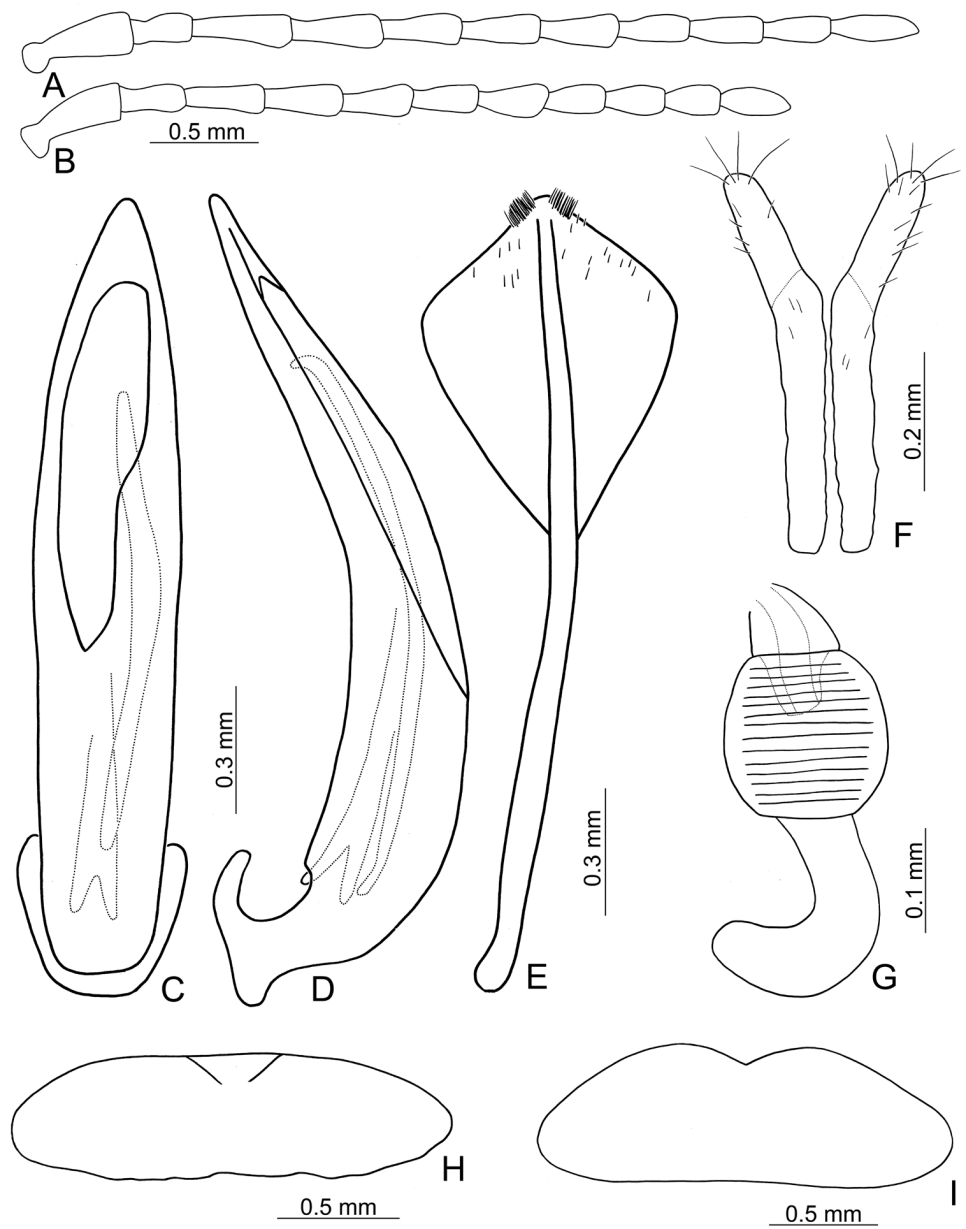
Diagnostic characters of *Lochmaeasmetanai* Kimoto. **A** Antenna, male **B** Antenna, female **C** Median lobe, dorsal view **D** Median lobe, lateral view **E** Abdominal ventrite VIII **F** Gonocoxae **G** Spermatheca **H** Abdominal ventrite V, male **I** Abdominal ventrite V, female.

**Figures 10.**
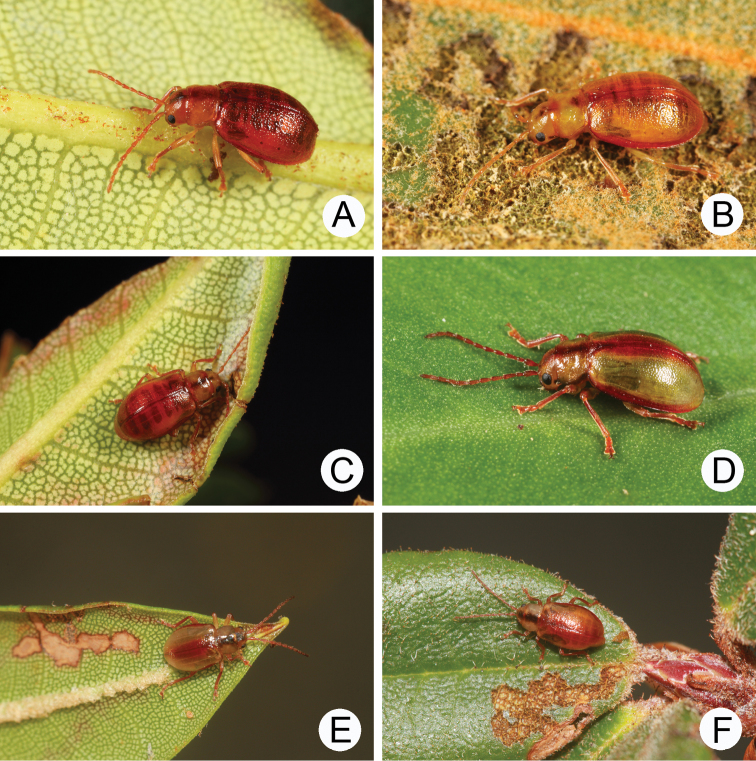
Ecological photography of *Lochmaea* species. **A***L.smetanai* Kimoto, from Hsuehshan **B** Same species, from Nanhutashan **C** Same species, from Hehuashan **D***L.cheni* Lee, sp. n., from Kuanshan Wind Gap **E***L.jungchani* Lee, sp. n., from Yushan West Peak **F** Same species, from Yushan East Peak.

Figs 8 , 9 , 10A–10C

Lochmaea smetanai Kimoto, 1996: 30.

#### Type material.

Holotype ♂ (NMNS), labeled: “TAIWAN Taichung Hsien / Hsuehshan, Hsuehshan / Main Peak 3650 m / 9.V.91 A. Smetana [T73] // Lochmaea / smetanai / Kimoto, n. sp. [h] / Det. S. Kimoto, 19 [p, w] // HOLOTYPE [p, r] // 2279-3 [p, w]”.

#### Other material examined

**(n = 233). Hualien**: 3♂♂, 4♀♀ (TARI), Chilai North Peak (奇萊北峰), 3600 m, 21.X.2017, leg. J.-C. Chen; **Miaoli**: 4♂♂, 10♀♀ (TARI), Chungpaping (中霸坪), 3300 m, 23.VI.2018, leg. J.-C. Chen; 4♂♂, 4♀♀ (TARI), Tsuichih Lodge (翠池山莊), 3550 m, 2.IX.2014, leg. J.-C. Chen; 19♂♂, 19♀♀ (TARI), same but with “leg. T.-H. Lee”; **Nantou**: 2♂♂, 3♀♀ (TARI), Chilai South Peak (奇萊南峰), 3350 m, 2.IX.2017, leg. J.-C. Chen; 6♂♂, 6♀♀ (TARI), Hehuanshan (合歡山), 3400 m, 26.VII.2014, leg. J.-C. Chen; 1♂, 4♀♀ (TARI), same locality, 30.VII.2014, leg. T.-H. Lee; 21♂♂, 13♀♀ (TARI), same but with “leg. C.-F. Lee”; 5♀♀ (TARI), same locality, 9.VIII.2014, leg. M.-H. Tsou; 3♂♂, 2♀♀ (TARI), Hehuan Eastern Peak (合歡東峰), 3420 m, 23.VII.2015, leg. J.-C. Chen; 2♂♂, 5♀♀ (TARI), same but with “22.VI.2016”; 7♂♂, 5♀♀ (TARI), Hehuan Western Peak (合歡西峰), 3145 m, 23.VI.2016, leg. J.-C. Chen; 5♂♂, 2♀♀ (TARI), Hsiaochilai (小奇萊), 3150 m, 23.IX.2016, leg. J.-C. Chen; 4♂♂, 3♀♀ (TARI), same but with “22.X.2016”; 1♂, 1♀ (TARI), Kunyang (昆陽), 3050 m, reared from larvae, 23.VI.2009, leg. C.-F. Lee; 1♂, 1♀ (NMNS), Yuanfeng (鳶峰), 2750 m, 7.VIII.-11.IX.2001, leg. C.-S. Lin & W.-T. Yang; 2♂♂, 1♀ (NMNS), same but with “16.X.-14.XI.2001”; 1♂ (NMNS), same but with “12.III.-9.IV.2002”; 1♀ (NMNS), same but with “9.VII.-13.VIII.2002”; 1♂ (NMNS), same but with “13.VIII.-10.IX.2002”; 1♂, 1♀ (NMNS), same but with “17.IV.-7.V.2003”; 2♂♂ (NMNS), same but with “11.VI.-8.VII.2003”; 1♂ (NMNS), same but with “4.XI.-15.XII.2003”; 1♂ (NMNS), same but with “5.X.-16.XI.2004”; 1♂ (NMNS), same but with “8.XI.-8.XII.2005”; 1♀ (NMNS), same but with “2–30.X.2007”; 1♀ (TARI), same locality, 29.VII.2014, leg. C.-F. Lee; 1♀ (TARI), same locality, 9.IX.2014, leg. C.-F. Lee; 3♂♂, 2♀♀ (TARI), same locality, 9.VIII.2014, leg. M.-H. Tsou; **Taichung**: 3♀♀ (TARI), Chungyangchienshan (中央尖山), 3705 m, 29.VII.2018, leg. J.-C. Chen; 1♂ (TARI), Hsuehshan (雪山), 3850 m, 1.IV.2010, leg. W.-B. Yeh; 1♂ (TARI), same but with “18.VI.2010”; 1♂, 1♀ (TARI), same but with “10.VI.2011”; 3♂♂, 1♀ (TARI), same locality, 3.IX.2014, leg. J.-C. Chen; 1♂, 2♀♀ (TARI), Nanhupeishan (南湖北山), 3536 m, 26.VII.2018; 7♂♂, 4♀♀ (TARI), Nanhutashan (南湖大山), 3700 m, 23.VII.2016, leg. J.-C. Chen; 3♂♂, 5♀♀ (TARI), Shengmacheng (審馬陣), 3200 m, 26.V.2018, leg. J.-C. Chen; 1♂ (TARI), same but with “26.VII.2018”.

#### Diagnosis.

*Lochmaeasmetanai* Kimoto cannot be distinguished from *L.jungchani* sp. n. based on external morphology but differs in the relatively broader median lobe, 5.7× longer than wide (Fig. 9C) (more slender median lobe in *L.jungchani* sp. n., 6.8× longer than wide (Fig. 13C)); longer elongate endophallic sclerite, 0.7× as long as median lobe (Fig. 9C) (shorter elongate endophallic sclerite in *L.jungchani* sp. n., 0.5× as long as median lobe (Fig. 13C)); apical margin of abdominal ventrite V in females with a median angular notch (Fig. 9I) (narrow notch margined with longitudinal ridges (Fig. 13I) in *L.jungchani* sp. n.).

#### Redescription.

Length 5.7–6.4 mm, width 2.9–3.5 mm. General color (Fig. 8A–C) reddish brown, but vertex and pronotum greenish brown, with median longitudinal dark band on pronotum, each elytron greenish brown except suture and lateral margins. Antennae filiform in males (Fig. 9A), length ratios of antennomeres I–XI 1.0 : 0.5 : 0.9 : 0.8 : 0.7 : 0.7 : 0.7 : 0.6 : 0.6 : 0.6 : 0.8, length to width ratios of antennomeres I–XI 2.6 : 2.0 : 3.1 : 2.8 : 2.7 : 2.6 : 2.6 : 2.5 : 2.6 : 2.5 : 3.3; a little smaller in females (Fig. 9B), length ratios of antennomeres I–XI 1.0 : 0.6 : 0.7 : 0.7 : 0.7 : 0.6 : 0.6 : 0.6 : 0.5 : 0.5 : 0.7, length to width ratios of antennomeres I–XI 2.7 : 2.4 : 2.6 : 2.5 : 2.2 : 2.3 : 2.3 : 2.1 : 2.0 : 2.0 : 2.5. Pronotum transverse, 1.5× wider than long, disc with dense, extremely coarse punctures, and one pair of lateral depressions; lateral margins strongly narrowed basally; margins concave basally and apically. Elytra longitudinal and broadly rounded, 1.4× longer than wide; disc with random, dense, and extremely coarse punctures. Apical margin of abdominal ventrite V in males straight, with median notch bearing short, oblique ridges at margin (Fig. 9H). Ventrite V in females with shallow, wide, median, angular notch (Fig. 9I). Median lobe (Fig. 9C, D) slender, 5.7× longer than wide, apically tapering from apical 1/3, parallel-sided from base to apical 1/3; opening elongate, located on right, starting from apical 1/7; in lateral view strongly curved, slightly oblique; internal sac with one elongate sclerite, 0.7× as long as median lobe, one additional sclerite located near base of elongate sclerites, base wide and bifurcate, apically membranous. Gonocoxae (Fig. 9F) elongate, separated, weakly sclerotized except apical parts; apical parts elongate, bearing small, scattered setae and four long setae at apices. Ventrite VIII (Fig. 9E) longitudinal and well sclerotized; apex rounded; abruptly broader at apical 1/5, with paired cluster of long setae near middle, disc bearing scattered, tiny setae along apical margin; spiculum long and narrow. Receptacle of spermatheca (Fig. 9G) strongly swollen; pump slender and strongly curved; proximal spermathecal duct deeply inserted into receptacle, broad but short.

#### Variability.

Some specimens have reduced punctation on the pronotum. Different individuals have different color patterns from brown to dark reddish brown (Fig. 8D–F).

#### Host plants.

Ericaceae: *Rhododendronpseudochrysanthum* Hayata (Fig. 10A–C).

#### Biology.

Some populations *Lochmaeasmetanai* Kimoto are sympatric with *L.lesagei* Kimoto or *L.tsoui* sp. n. when microhabitats are stable at high altitudes (at or above 3000 m). For example, larvae of this species were collected in Kunyang (昆陽) (3050 m) with those of *L.tsoui* sp. n. (see biology to *L.tsoui* sp. n. for details). Adults might be long-lived, based on their occurrence in the field from April to December.

#### Distribution.

Central Taiwan, including Miaoli, Taichung, Nantou, and Hualien Counties (Fig. 5B).

### Lochmaea cheni sp. n.

Taxon classificationAnimaliaColeopteraChrysomelidae

http://zoobank.org/7C059985-0473-4858-BC5F-06346B3A9E5F

**Figures 11.**
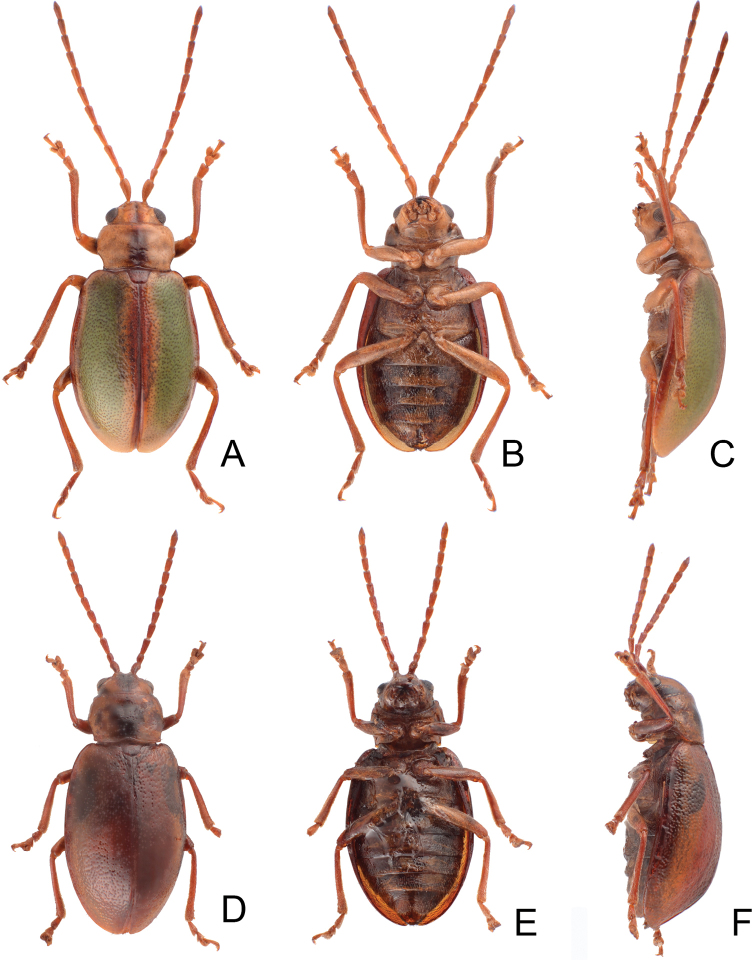
Habitus of *Lochmaea* species. **A***L.cheni* Lee, sp. n., male, dorsal view **B** Ditto, ventral view **C** Ditto, lateral view **D***L.jungchani* Lee, sp. n., female, dorsal view **E** Ditto, ventral view **F** Ditto, lateral view.

**Figures 12.**
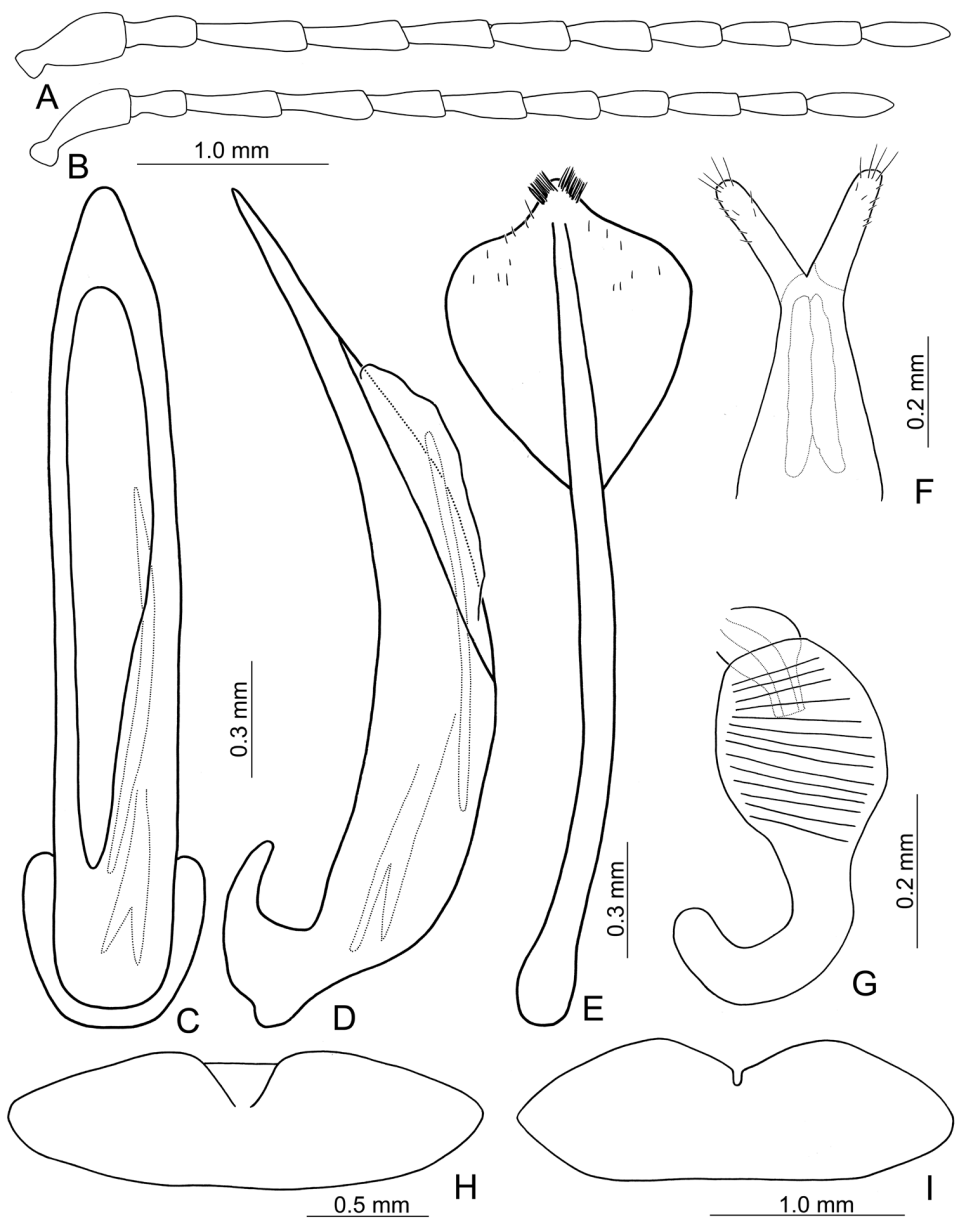
Diagnostic characters of *Lochmaeacheni* Lee, sp. n. **A** Antenna, male **B** Antenna, female **C** Median lobe, dorsal view **D** Median lobe, lateral view **E** Abdominal ventrite VIII **F** Gonocoxae **G** Spermatheca **H** Abdominal ventrite V, male **I** Abdominal ventrite V, female.

Figs 10D , 11A–11C , 12

#### Type material

**(n = 64).** Holotype ♂. **Kaoshiung**: Kuanshan Wind Gap (關山啞口), 2700 m, 30.VII.2015, leg. C.-F. Lee. Paratypes. 39♂♂, 18♀♀, same data as holotype; **Pingtung**: 1♀ (TARI), Peitawushan (北大武山), 3050 m, 13.X.2018, leg. J.-C. Chen; **Taitung**: 3♂♂, 2♀♀ (TARI), Hsiangyangshan (向陽山), 3600 m, 19.IX.2014, leg. J.-C. Chen; 1♂ (TARI), same but with “6.VIII.2015.

#### Diagnosis.

**Figures 13.**
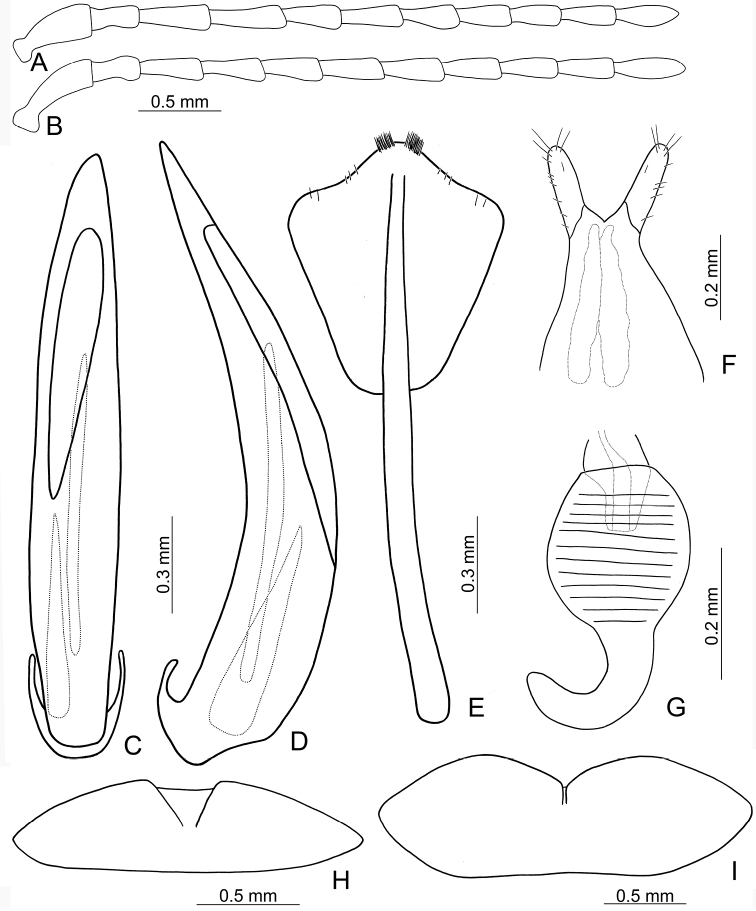
Diagnostic characters of *Lochmaeajungchani* Lee, sp. n. **A** Antenna, male **B** Antenna, female **C** Median lobe, dorsal view **D** Median lobe, lateral view **E** Abdominal ventrite VIII **F** Gonocoxae **G** Spermatheca **H** Abdominal ventrite V, male **I** Abdominal ventrite V, female.

*Lochmaeacheni* sp. n. is easily distinguished from other members of the species group by the green elytra (Fig. 11A–C) (entirely reddish brown or yellowish brown elytra in others (Figs 8, 11D–F)), parallel-sided median lobe (Fig. 12C) (tapering median lobe (Figs 9C, 13C) in others) and opening located more posteriorly, and apical margin of abdominal ventrite V in females bearing a narrow, shallow notch (Fig. 12I) (angular notch (Fig. 9I) in *L.smetanai*; narrower notch margined with longitudinal ridges (Fig. 13I) in *L.jungchani* sp. n.).

#### Description.

Length 6.2–7.2 mm, width 3.3–3.9 mm. General color (Fig. 11A–C) yellowish brown to reddish brown; vertex and pronotum with median longitudinal dark band; each elytron green but with wide yellowish brown band along suture and lateral margin. Antennae filiform in males (Fig. 12A), length ratios of antennomeres I–XI 1.0 : 0.6 : 1.0 : 0.9 : 0.8 : 0.7 : 0.7 : 0.6 : 0.6 : 0.6 : 0.8, length to width ratios of antennomeres I–XI 2.4 : 2.2 : 3.7 : 3.5 : 3.1 : 2.6 : 2.7 : 2.6 : 2.7 : 3.0 : 3.6; similar in females (Fig. 12B), length ratios of antennomeres I–XI 1.0 : 0.5 : 0.8 : 0.8 : 0.7 : 0.7 : 0.7 : 0.6 : 0.6 : 0.6 : 0.8, length to width ratios of antennomeres I–XI 3.0 : 2.1 : 3.4 : 3.4 : 2.7 : 2.8 : 2.6 : 2.8 : 3.0 : 3.0 : 3.7. Pronotum transverse, 1.6× wider than long, disc with sparse, extremely coarse punctures, and one pair of lateral depressions; lateral margins strongly narrowed basally; margins concave basally and apically. Elytra longitudinal with lateral margins broadly rounded, 1.3–1.4× longer than wide; disc bearing random, dense, coarse punctures. Apical margin of abdominal ventrite V in males with median notch, bearing short, oblique ridges at margin, weakly concave between ridges. Ventrite V in females medially depressed, with narrow, shallow notch at middle. Median lobe (Fig. 12C, D) slender, 6.8× longer than wide, apically tapering from apical 1/7, parallel-sided from base to apical 1/7; opening elongate, starting from apical 1/5 located on right; in lateral view strongly curved, slightly oblique; internal sac with one elongate sclerite, 0.5× as long as median lobe, one additional sclerite located near base of elongate sclerites, base wide and bifurcate, apically membranous. Gonocoxae (Fig. 12F) elongate, membranous except apical parts, with one pair of weakly sclerotized, elongate sclerites at base; apical parts elongate, bearing tiny, scattered setae and four long setae at apices. Ventrite VIII (Fig. 12E) longitudinal and well sclerotized; apex rounded; abruptly broader at apical 1/5, with paired cluster of long setae near middle, disc bearing scattered, tiny setae along apical margin; spiculum long and narrow. Receptacle of spermatheca (Fig. 12G) strongly swollen; pump slender and strongly curved; proximal spermathecal duct deeply inserted into receptacle, broad but short.

#### Variability.

Some specimens have reduced punctures on the pronotum. Few specimens have yellowish brown elytra but suture and lateral margin reddish brown.

#### Host plants.

Ericaceae: *Rhododendronpseudochrysanthum* Hayata (Fig. 10D).

#### Biology.

Unknown. Adults are active from July to September.

#### Etymology.

This new species is named after Mr Jung-Chan Chen, a member of the TCRT and the first to collect this new species.

#### Distribution.

High mountains along South Cross-Island Highway (Kaoshiung and Taitung Counties).

### Lochmaea jungchani sp. n.

Taxon classificationAnimaliaColeopteraChrysomelidae

http://zoobank.org/80392637-C8B1-4DE4-B3BE-080509E7E975

Figs 10E, F , 11D–F , 13

#### Type material

**(n = 33).** Holotype ♂ (TARI): **Chiayi**: Yushan East Peak (玉山東峰), 3869 m, 20.IX.2018, leg. J.-C. Chen. Paratypes. 7♂♂, 8♀♀ (TARI), same data holotype; 6♂♂, 5♀♀ (TARI), Yushan North Peak (玉山北峰), 3858 m, 20.IX.2018, leg. J.-C. Chen; 2♂♂ (TARI), Yushan West Peak (玉山西峰), 3518 m, 19.IX.2018, leg. J.-C. Chen; 3♀♀ (TARI), Yushan Main Peak (玉山主峰), 3950 m, 17.VIII.2017, leg. J.-C. Chen; 1♂ (TARI), Paiyun Lodge (排雲山莊), 3400 m, 24.X.2017, leg. J.-C. Chen.

#### Diagnosis.

*Lochmaeajungchani* sp. n. cannot be distinguished from *L.smetanai* Kimoto based on external morphology but differs with the relatively slender median lobe, 6.8× longer than wide (Fig. 13C) (broader median lobe in *L.semtanai*, 5.7× longer than wide (Fig. 9C)); shorter elongate endophallic sclerite, 0.5× as long as median lobe (Fig. 13C) (longer elongate endophallic sclerite in *L.smetanai*, 0.7× as long as median lobe (Fig. 9C)); apical margin of abdominal ventrite V in females with narrow notch margined with longitudinal ridges (Fig. 13I) (angular notch in *L.smetanai* (Fig. 9I)).

#### Description.

Length 5.5–6.5 mm, width 2.8–3.3 mm. General color (Fig. 11D–F) yellowish brown to reddish brown; vertex and pronotum with median longitudinal dark stripe. Antennae filiform in males (Fig. 13A), length ratios of antennomeres I–XI 1.0 : 0.6 : 0.9 : 0.9 : 0.7 : 0.7 : 0.7 : 0.7 : 0.6 : 0.7 : 0.8, length to width ratios of antennomeres I–XI 2.1 : 2.1 : 2.9 : 3.2 : 2.5 : 2.6 : 2.5 : 2.6 : 2.5 : 2.8 : 3.1; similar in females (Fig. 13B), length ratios of antennomeres I–XI 1.0 : 0.5 : 0.7 : 0.7 : 0.6 : 0.7 : 0.6 : 0.6 : 0.6 : 0.6 : 0.8, length to width ratios of antennomeres I–XI 3.3 : 2.0 : 2.7 : 2.7 : 2.5 : 2.7 : 2.6 : 2.7 : 2.8 : 2.8 : 3.3. Pronotum transverse, 1.6× wider than long, disc with sparse, extremely coarse punctures, and one pair of lateral depressions; lateral margins strongly narrowed basally; margins concave basally and apically. Elytra longitudinal and broadly rounded, 1.4× longer than wide; disc with random, dense, and extremely coarse punctures. Apical margin of abdominal ventrite V in males rounded, with median notch bearing short, oblique ridges at margin, weakly depressed between ridges. Ventrite V in females medially depressed, with narrow notch margined with longitudinal ridges at middle. Median lobe (Fig. 13C, D) extremely slender, 6.8× longer than wide, apically tapering from middle, parallel-sided from base to middle; opening elongate, located on right, starting from apical 1/6; in lateral view strongly curved, slightly oblique; internal sac with one elongate sclerite, 0.5× as long as median lobe, one additional sclerite located near base of elongate sclerites, base wide, apically tapering. Gonocoxae (Fig. 13F) elongate, membranous except apical parts, with one pair of weakly sclerotized, elongate sclerites at base; apical parts elongate, bearing tiny, scattered setae and four long setae at apices. Ventrite VIII (Fig. 13E) longitudinal and well sclerotized; apex rounded; abruptly broader at apical 1/5, with paired cluster of long setae near middle, disc bearing scattered, tiny setae along apical margin; spiculum long and narrow. Receptacle of spermatheca (Fig. 13G) strongly swollen; pump slender and strongly curved; proximal spermathecal duct deeply inserted into receptacle, broad but short.

#### Host plant.

Ericaceae: *Rhododendronpseudochrysanthum* Hayata (Fig. 10E, F).

#### Biology.

Unknown. Adults are active in the field from August to October.

#### Etymology.

This new species is named after Mr. Jung-Chan Chen, a member of the TCRT and the first to collect this new species.

#### Distribution.

Yushan and surrounding areas (Chiayi County).

## Discussion

Taiwanese species of *Lochmaea* are characterized by the uniform first tarsomere of the metatarsus (enlarged first tarsomere of male metatarsus in others), last abdominal ventrite in males, and median lobes (both characters are very complex and diagnostic for others). Species richness of the wingless *Lochmaeasmetanai* group is less than that of any other wingless galerucines in Taiwan, including ten species in *Paraplotes* Laboissière (Lee 2015), five species in *Sikkimia* Duvivier (Lee and Bezděk 2016), and six species in *Shairella* Chûjô (Lee and Beenen 2017). Moreover, the aedeagi of congeners are more similar to each other than in other genera. Both features imply that reduction of hind wings is a recent evolutionary event. Although male genitalic characters are less diagnostic, some female genitalic characters are useful in species delimitation, including the shapes of abdominal ventrites V and VIII. Abdominal ventrites VIII in females are characteristic in that they are well sclerotized, subapically expanding, and with sides curving inwards. They appear to replace the base of the gonocoxae functionally.

**Figures 14.**
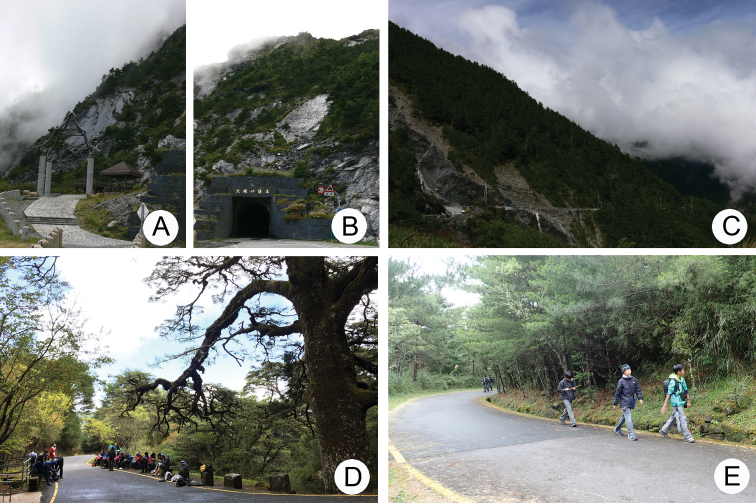
Microhabitats. **A** Kuanshan Wind Gap **B** Same locality, different angle **C** Same locality, different angle **D** Tatachia **E** Same locality, different angle.

Members of the winged *Lochmaealesagei* group usually inhabit mountains above 2000 m, but some populations occur at less than 1500 m in northern Taiwan. They seem to occur in alpine environments only when microhabitats are stable. For example, most larvae collected from Kunyang (昆陽, 3050 m) belong to *L.lesagei*. By contrast, adults and larvae of *L.smetanai* group (wingless) are restricted to alpine habitats above 3000 m. One exception is Kuanshan Wind Gap (關山啞口, 2700 m) (Fig. 14A–C) where it is so windy that it takes on “alpine” characteristics although the altitude is below 3000 m. This microhabitat is suitable for a wingless population (*L.cheni* sp. n.) where more than 50 specimens of *L.cheni* sp. n. were collected from three plants by beating. For comparison, Tatachia (塔塔加, 2600 m) is almost as high as Kuanshan Wind Gap, but the microhabitats are stable (Fig. 14D, F). Although adults of *Lochmaea* were common there, all were winged (*L.lesagei*). Other wingless galerucines in Taiwan inhabit stable, mid-altitude habitats. These include members of *Sikkimia* Duvivier (Lee and Bezděk 2016) and *Sharella* Chûjô (Lee and Beenen 2017). These observations suggest that distributions of winged and wingless species of *Lochmaea* are the only chrysomelids in Taiwan that fit expectations of preferred habitats in brachyelytrous species (Beenen and Jolivet 2008).

## Supplementary Material

XML Treatment for Lochmaea lesagei

XML Treatment for Lochmaea tsoui

XML Treatment for Lochmaea smetanai

XML Treatment for Lochmaea cheni

XML Treatment for Lochmaea jungchani
